# What do clinicians need to watch for with direct‐acting antiviral therapy?

**DOI:** 10.1002/jia2.25076

**Published:** 2018-04-10

**Authors:** Alessio Aghemo, Lionel Piroth, Sanjay Bhagani

**Affiliations:** ^1^ Department of Biomedical Sciences Humanitas University Pieve Emanuele Milan Italy; ^2^ Division of Internal Medicine and Hepatology Humanitas Clinical and Research Center Rozzano Milan Italy; ^3^ Infectious Diseases Department University Hospital INSERM Dijon France; ^4^ Department of Infectious Diseases/HIV Medicine Royal Free London Foundation Trust Research Department of Infection UCL London UK

**Keywords:** Hepatitis, HCV, Baseline, Safety, DAA, Coinfection

## Abstract

**Introduction:**

The introduction of drugs targeting the virus replication cycle has revolutionized treatment of chronic hepatitis C virus. These drugs, called direct‐acting antivirals, have brought about extremely high rates of virological cure and have increased the number of patients who can receive treatment due to the lack of absolute contraindications. A combination of different classes of direct‐acting antivirals is the current standard of care. Although treatment administration and monitoring has been simplified in recent years, it is still relatively complex and mostly in the hands of specialists. Several factors must be assessed before starting treatment to maximize efficacy and minimize side effects of treatment. In this review, we describe the factors that impact on the efficacy and safety of antiviral treatment for hepatitis C and provide clear recommendations for clinicians prescribing direct‐acting antivirals.

**Methods:**

We reviewed literature to define best practice, based on factors associated with treatment efficacy and safety data to recommend treatment options, baseline and on‐treatment assessments. The review included searches in PubMed, and the abstracts presented at the International Liver Congress TM and The Liver Meeting TM between January 2013 and September 2017.

**Results:**

Clinical features that must be assessed before starting treatment include virological factors, such as hepatitis C virus genotype, HIV and hepatitis B coinfection and host factors, such as concomitant medications, liver disease stage and renal function.

**Conclusions:**

Patients who start antiviral treatment for chronic hepatitis C require a thorough clinical evaluation. There is a need for assessing factors that impact on the treatment schedule and duration or affect the pharmacokinetics of direct‐acting antivirals.

## 1 Introduction

Treatment of chronic hepatitis C virus (HCV) has been revolutionized by the introduction of drugs, called direct‐acting antivirals (DAAs) 1, 2, which target the virus replication cycle. These drugs target key steps in HCV replication: polyprotein cleavage by the NS3 protease (protease inhibitors, PIs); HCV RNA strain synthesis (NS5B polymerase inhibitors of two sorts; nucleoside analogues, NA, and non‐nucleosides); and stabilization of the replication complex and viral release (NS5A inhibitors) 3, 4. A combination of different classes of DAAs is essential to obtain viral replication suppression and ultimately viral clearance.

Sustained virological response, defined as serum HCV RNA below the limit of detection 12 weeks after the end of therapy (SVR 12), is the goal of treatment. SVR 12 is a marker of virological and clinical cure as patients who reach this endpoint show decreased mortality and improved quality of life 5. Until 2013, treatment of HCV was based on pegylated interferon, a drug that was introduced in 1986 and achieved SVR rates in the 50% range while also being associated with significant side effects 5. Currently, SVR 12 rates in the 90% to 95% range are achievable in most patient populations 5, 6.

While DAAs have greatly simplified the management of patients with chronic HCV infection, treatment of HCV is still relatively complex and mostly in the hands of specialists 7. Although economic considerations are at least in part responsible for this restriction, there are still factors that modify the efficacy and safety of DAAs; these must be assessed before staring treatment. The therapeutic drug development process in HCV has been completed, and the only possible improvements to currently available DAAs will be in the form of optimization of treatment in groups where there are still gaps in knowledge. It is therefore important that knowledge around DAA‐based HCV treatment is widely disseminated while we move towards simplification of pre‐, on‐ and post‐treatment monitoring and make treatments available widely outside of specialist care. In this review article, we will summarize the current knowledge on what clinicians should assess before, during and after DAA‐based therapy 8, 9.

## 2 Methods

We conducted a PubMed search looking for articles assessing factors associated with DAA treatment efficacy and safety, restricted to articles published between January 2013 and September 2017. We also analysed the abstracts presented at the International Liver Congress™ and The Liver Meeting™. We used permutations of the following search terms “Hepatitis C treatment” “Direct Acting Antivirals” “Complications” “Monitoring” and “HIV.” We restricted the search to abstracts/articles published in English.

## 3 Results

### 3.1 Pre‐treatment assessment

**Figure 1:**
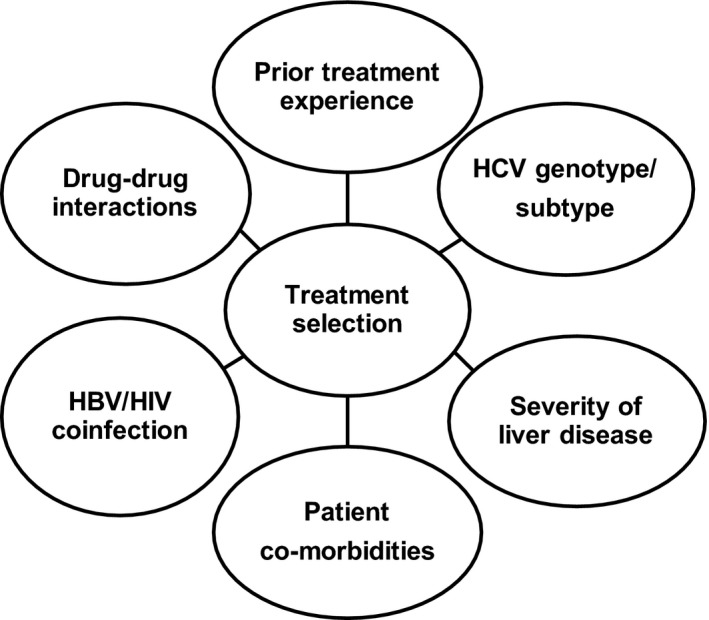
Pre‐treatment variables that must be assessed before starting DAA therapy.

Before starting DAA therapy in patients with HCV infection, pre‐treatment assessment should be aimed at determining factors that modify the safety and the efficacy of DAAs 5. Factors that are known modifiers of efficacy and safety include: HCV genotype; liver disease severity; comorbid conditions that include coinfection with HBV, HIV and renal impairment; and concomitant medications. These factors determine the optimal treatment choice at the individual level (Figure 1 and Table 1).

**Table 1:** Interpretation of pre‐treatment assessment in DAA candidates

Variable	Test	Interpretation
HCV genotype	Commercial assay using the sequence of the 5′untranslated region plus a portion of another genomic region, generally the core‐coding or the NS5B‐coding regions	Choose DAA regimen for specific HCV genotype following international guidelines
Disease stage	Transient elastography APRI: [{AST (IU/L)/AST_ULN (IU/L)} × 100]/ platelet count (109/L) FIB‐4: age (yr) × AST(IU/L)/platelet count (109/L × [ALT(IU/L)1/2] Liver biopsy	Cirrhosis: Plan surveillance schedule and assess complete liver function. Choose DAA regimen schedule based on fibrosis stage.
Liver function	Child‐Pugh‐Turcotte Score (Albumin, INR, Bilirubin, Ascites, encephalopathy)	CPT = A6 Prefer DAAs not including protease inhibitors. CPT >A6 Protease inhibitors must be avoided
Kidney function	Assess eGFR (Ckd‐Epi, Cockcroft‐Gault formula, MDRD)	eGFR <30 ml/min/m^2^ avoid sofosbuvir‐based regimens.
Concomitant medications	Assess comorbidities and concomitant medications (focus on immunosuppressant, cardiovascular and lipid‐lowering drugs). Warnings for PPIs and HIV medications	Check international guidelines and http://www.hep-druginteractions.org
HBV status	HBsAg, Anti‐HBs, Anti‐HBc. If HBsAg + check HBV DNA, HBeAg and anti‐HBeAg	HBsAg negative, anti‐HBc positive: Monitor and test for HBV reactivation in case of ALT elevation (check every 4 weeks). HbsAg‐positive patients fulfilling the standard criteria for HBV treatment should receive treatment following international guidelines. HBsAg‐positive patients not meeting HBV treatment criteria should be considered for concomitant nucleos(t)ide analogue prophylaxis until week 12 post DAA, and monitored closely.

### 3.2 HCV genotype determination

HCV circulates in seven different genotypes worldwide 10. HCV genotype does not play a major role as a modifier of the natural course of the disease, but has a dramatic influence on the efficacy of pegylated interferon‐based regimens 5. Not all DAAs are affected in the same way by HCV genotype as some are pangenotypic while others are restricted in efficacy to specific HCV genotypes. Moreover, in patients with HCV genotype 1 infection, there is a need for subtyping. This is because subtype 1a, with a lower barrier to genotypic resistance with first‐ and second‐generation PIs, requires extended treatment duration and/or the addition of ribavirin with the combination of grazoprevir/elbasvir, or use of sofosbuvir/ledipasvir or ritonavir‐boosted paritaprevir/ombitasvir plus dasabuvir 5, 9.

The arrival of the next‐generation potent DAA combinations with pangenotypic activity – sofosbuvir/velpatasvir (SOF/VEL), glecaprevir/pibrentasvir (G/P) and sofosbuvir/velpatasvir/voxilaprevir (SOF/VEL/VOX) – limits the need for HCV genotyping and subtyping. However, before abandoning HCV genotyping, we should bear in mind that access to pangenotypic regimens is not universal and optimal treatment schedules with G/P, SOF/VEL and SOF/VEL/VOX still varies according to HCV genotype 9. With these three pangenotypic regimens, there is certainly a need for identifying genotype 3 HCV (G3) patients. G/P requires 16 weeks of treatment in G3 patients who have failed a previous course of treatment, while SOF/VEL requires the addition of ribavirin in G3 treatment‐experienced patients and cirrhotic patients 5, 9.

SOF/VEL/VOX is less impacted by HCV genotype, as 12 weeks of treatment with this regimen are able to achieve >95% SVR rates independently from HCV genotype, previous DAA failure or fibrosis stage 9 However, identifying patients with G3 infection is important since a short treatment duration of 8 weeks is sufficient in G3 treatment‐naïve patients with cirrhosis 9.

The European Association for the Study of the Liver (EASL) recommends: “Genotyping/subtyping should be performed with an assay that accurately discriminates subtype 1a from 1b, i.e. an assay using the sequence of the 5′untranslated region plus a portion of another genomic region, generally the core‐coding or the NS5B‐coding regions.” 5. Other tests based on assessment of only one region of the HCV genome have been shown to be less accurate for the identification of genotype 1 subtype and thus should not be routinely performed if a DAA that is subtype susceptible must be used 5.

### 3.3 Disease severity

Identifying patients with cirrhosis or advanced fibrosis affects the choice of the treatment regimen and the post‐treatment prognosis, as well as the post‐treatment follow‐up schedule 5. In the past, assessment of disease severity was based on histological fibrosis staging by liver biopsy; this procedure is now unnecessary due to the risk of serious side effects, as well as the development of accurate non‐invasive methods. Considerable evidence supports the use of non‐invasive methods as first‐line modality for liver disease staging 11. Liver stiffness measurement can assess liver fibrosis and the presence of portal hypertension in patients with chronic hepatitis C if consideration is given to factors that may adversely affect its performance, such as obesity, fasting status, other causes of liver disease and alanine aminotransferase (ALT) values 11.

Transient elastography values <10 KPa can rule out the presence of advanced fibrosis and values >12.5 to 14 KPa are suggestive of the presence of advanced fibrosis/cirrhosis. Screening for oesophageal varices is recommended in patients with elastography values >20 KPa and/or platelet count <150,000 μL 12. Transient elastography is reproducible and has a high level of acceptability by patients, but is still not readily available worldwide and requires investment in expensive equipment and trained operators. In resource‐limited settings, or where transient elastography is not available, World Health Organization (WHO) guidelines for HCV management 13 recommend using serum tests, such as the APRI, or FIB4 scores, which measure indirect markers of fibrosis, for example, ALT, aspartate aminotransferase (AST) and platelet count to assess fibrosis stage. All non‐invasive methods to assess fibrosis stage must be performed before starting treatment. Their interpretation following achievement of an SVR can be tricky as, for example, early post‐treatment reduction in liver stiffness may reflect resolution of liver inflammation 14 and could lead to misclassification of patients with advanced disease.

In patients with cirrhosis, precise definition of liver function to assess the prognosis of the disease is mandatory. The Child‐Pugh‐Turcotte score (CPT), which requires measurement of five clinical and laboratory variables (prothrombin time; albumin values; presence and degree of encephalopathy; presence and degree of ascites; and bilirubin levels), should be performed before the start of any DAA treatment. The CPT score divides patients into 3 classes: A when the score is 5 or 6, B when the score is 7 to 9, and C when it is 10 to 15. In patients with CPT A5, all DAAs can be safely administered. By contrast, in patients with CPT B and C, any DAA regimen that includes a NS3 protease inhibitor must be avoided because PIs are extensively metabolized in the liver by the CYP3A4 family of enzymes. Thus, in the presence of impaired liver function (CPT B and C) a >100 fold increase in the serum levels of PIs can be seen. The use of PIs in patients with CPT B and C class has resulted in cases of further liver function deterioration, hepatic decompensation and death 5, 9. No firm recommendation can be given in patients with CPT A6, where PIs appear to be safe. However, given that these patients might be unstable and CPT class might transition rapidly from A to B, it is our opinion that PIs should not be the preferred option in this group of patients.

### 3.4 Renal function

From a pathophysiological point of view, there is a direct link between chronic HCV infection and kidney impairment 15. Not only can HCV lead to the development of cryoglobulinemic glomerulonephritis, but it can also cause kidney impairment due to direct inflammation. Moreover, diabetes is more prevalent in HCV patients due to a direct link between viral infection and development of insulin resistance 15.

Overall, HCV infection is associated with a 43% increase in the risk of developing chronic kidney disease (CKD) 15. Kidney function can be measured easily by calculating the estimated glomerular filtration rate in ml/min (eGFR). With respect to DAAs, it is mandatory to identify those patients with a CKD stage 4‐5, i.e., eGFR <30 mL/min/1.73 m^2^, as sofosbuvir should be used with caution in this subgroup 5. Indeed, approximately 80% of sofosbuvir is renally excreted. The majority of the sofosbuvir dose recovered in urine is the dephosphorylation‐derived nucleoside metabolite GS‐331007 (78%), while 3.5% is recovered as sofosbuvir. Renal clearance is the major elimination pathway for GS‐331007 with a large part actively secreted 5. Based on these findings, the current EASL recommendations for the treatment of HCV state that “no sofosbuvir dose recommendation can be given for patients with severe renal impairment (eGFR <30 mL/min/1.73 m^2^) or with end‐stage renal disease due to higher exposures (up to 20‐fold) of GS‐331007”. This limitation currently affects only a small group of people (0.3% of the general population), but still accounts for a significant knowledge gap in patients who have previously failed a PI‐containing DAA regimen where the optimal retreatment option is the combination of SOF/VEL/VOX 9.

Preliminary real‐life data where sofosbuvir was given to patients with CKD stage 4‐5 have provided conflicting results with most not showing an increased rate of side effects 16. However, in the Target report by Saxena and colleagues, the CKD stage 4‐5 patients who received sofosbuvir‐based regimens showed a 30% rate of eGFR deterioration and an 18% incidence of serious adverse events, which were both statistically higher than that observed in patients with preserved kidney function 17. For this reason, caution is warranted in using sofosbuvir in CKD stage 4‐5 patients, and a thorough risk/benefit analysis should be performed before contemplating use 5, 9, 18.

For DAA‐naïve patients with CKD stage 4‐5, DAA regimens not based on NS5B polymerase inhibitors, such as paritaprevir/ombitasvir/dasabuvir, grazoprevir/elbasvir and glecaprevir/pibrentasvir, are the preferred treatment options that have proven safety and efficacy in phase III clinical trials, as well as in real‐life cohorts 9. The only drawback is that these regimens include a PI, which is unsafe and not recommended for patients with decompensated cirrhosis. An HCV patient with stage 4‐5 CKD and decompensated liver disease is potentially untreatable with this option. Instead, if indicated, kidney and liver transplantation should be prioritized.

### 3.5 Hepatitis B virus coinfection

Although HCV and HBV share ways of transmission, coinfection with both viruses is generally rare. Epidemiological studies report a prevalence of HBV/HCV coinfection in the 0.2% to 1.9% range, with higher rates in Eastern Asia 19. From a clinical standpoint, HBV/HCV coinfection is associated with faster disease progression, higher rates of liver cancer and reduced survival. Most HBV/HCV‐coinfected patients will show active HCV replication with related liver damage and a concomitant HBeAg negative chronic hepatitis state (HBsAg positivity, HBeAg negative, HBV DNA <2000 IU/mL and lack of HBV‐induced liver damage) 20, 21, 22. This is the direct consequence of negative reciprocal interaction between HBV and HCV replication. Several reports in the pegylated interferon era showed that achievement of an SVR could derange this balance leading to HBV reactivation once HCV was cleared 19, 20, 23.

The same was reported in HBV/HCV‐coinfected patients receiving DAAs. Following the first FDA warning of 29 reported cases of HBV reactivation in DAA post‐marketing analysis, several large cohort studies (but not all) have reported cases of reactivation in HBV/HCV‐coinfected patients receiving DAAs with similar incidence to that reported with pegylated interferon‐based treatments 24. From 377 HBV/HCV‐coinfected patients, Belperio and colleagues reported HBV reactivation in eight patients (2%) 25. Similarly, in a large systematic analysis of 1185 patients, the cumulative incidence of HBV reactivation was 14.1% in HBsAg‐positive patients treated with DAAs 26.

Most importantly there is a need to assess the risk of reactivation in patients with isolated anti‐HBc serological profile (HBsAg negative, anti‐HBc positive), which represent more than 1 billion people worldwide 19. Current evidence does not show a major risk of reactivation in patients with positive HBc antibodies, with the few cases reported in the literature being biased by confounding factors, such as HIV coinfection, concomitant use of immunosuppressive therapy or concomitant extra‐hepatic malignancies 19, 27.

Therefore, all patients starting DAAs need to be evaluated for HBV coinfection (HBsAg, anti‐HBs, anti‐HBc). For HBsAg‐positive patients, definition of disease state is essential to understand the need for anti‐HBV treatment.

The most recent EASL HBV clinical practice guidelines state that 19:

1. Patients fulfilling the standard criteria for HBV treatment should receive anti‐HBV active nucleoside analogue (NA) treatment.
2. HBsAg‐positive patients undergoing DAA therapy should be considered for concomitant anti‐HBV NA prophylaxis until week 12 post DAA, and monitored closely.
3. HBsAg‐negative, anti‐HBc‐positive patients undergoing DAA should be monitored (ALT testing every 2 to 4 weeks) and tested for HBV reactivation in case of ALT elevation.

### 3.6 HIV/HCV coinfection

Since HIV and HCV share the same routes of transmission, HIV/HCV coinfection is not uncommon 28. Although use of combination antiretroviral therapy ameliorates some of the risk of accelerated hepatic fibrosis and clinical decompensation, this does not revert back to the levels seen in HCV monoinfected patients 29. This led to calls for wide access to anti‐HCV therapy, even though the response rates following conventional interferon‐based therapy were lower in coinfected patients than in HCV‐monoinfected patients 30. DAA‐based therapy has changed this as registration trials and real‐life cohorts suggest that HCV clearance rates are equivalent among HIV/HCV‐coinfected individuals and HCV monoinfected patients 31.

For all these reasons, access by HIV/HCV‐coinfected patients to all DAA therapy has been prioritized in many countries, whatever these patients’ fibrosis stage. There are, however a number of important drug‐drug interaction considerations to take into account before choosing an appropriate DAA regimen; this applies not only to antiretroviral therapy, but also to frequently prescribed co‐medications for the management of multiple comorbidities in this group of patients 5, 9, 32. Important drug‐drug interactions may not just be related to cytochrome P450 isoenzymes, but are also associated with a myriad of drug‐transporter proteins in the gastrointestinal tract, liver and the kidneys 32.

Indeed, interactions via renal drug transporters with DAAs, such as ledipasvir or velpatasvir, may increase the risk of renal impairment when combined with tenofovir DF 5, 9.

### 3.7 Drug‐drug interactions

Numerous and complex drug‐drug interactions (DDIs) are possible with the HCV DAAs 33. Once taken orally, DAAs must be absorbed and enter the blood circulation, a process regulated by gastric pH and gut transporters. Most of the DAAs will then be metabolized in the liver by the cytochrome P450 family of enzymes and then excreted either in the bile or by the kidney 5. DAAs can be excreted from enterocytes to the gut lumen or from hepatocytes into the biliary system by proteins, such as P‐glycoprotein 1 (P‐GP1) or breast cancer‐resistance protein (BCRP). These proteins and the gastric pH, as well as enzymes from the cytochrome P450 family, can be induced or inhibited by other concomitant drugs that the patients might be taking. Therefore, the potential for drug‐drug interactions is present in all patients planned for treatment with DAAs. This requires a thorough DDI risk assessment prior to starting therapy and before starting other medications during treatment. Data on potential DDIs can be found on the prescribing information for each DAA; a useful Internet resource is http://www.hep-druginteractions.org, where recommendations are regularly updated.

An exact list of all DDIs is beyond the scope of this review. However, clinicians should be aware of potential DDIs with anti‐HIV drugs, as we have highlighted. Similarly, commonly prescribed drugs, such as proton pump inhibitors (PPIs), might lower the efficacy of some DAAs. PPIs may be associated with reduced efficacy of sofosbuvir/ledipasvir 34. Velpatasvir is also impacted by gastric pH; for this reason, PPIs should be taken four hours after velapatsvir 5, 9. Other groups of patients that require careful evaluation of DDIs due to high risk of clinically significant interactions are recipients of organ transplantation and patients taking antiarrhythmic and/or antiplatelets and/or lipid‐lowering medications 5, 9.

However, DDIs are rarely a reason for not starting DAA treatment since therapeutic alternatives are usually possible and given the short duration of DAA‐based therapy for HCV, many DDIs can be overcome or circumvented.

### 3.8 On‐treatment monitoring

Treatment monitoring is aimed at assessing efficacy, safety and DDIs. Treatment monitoring was key during pegylated interferon‐based regimens as rules for treatment duration and treatment stopping were defined by HCV RNA on treatment kinetics, and haematological and systemic side effects were common 35. Given the high SVR rates achieved with DAA combinations and the current lack of a response guided treatment algorithm, monitoring of DAA treatment efficacy can be achieved by measuring HCV RNA at baseline and 12 weeks after the end of therapy to assess SVR12 5, 36. In all cases, HCV RNA testing should be performed with a real‐time PCR‐based assay with a lower limit of detection of ≤15 IU/mL 5.

Monitoring for side effects is also of little to no practical use as new DAA regimens are generally well tolerated with less than 1% of patients discontinuing treatment for side effects or reporting serious adverse events 5, 9. However, monthly assessment of liver function status is necessary in patients with advanced liver disease. Kidney function should also be assessed monthly in patients with CKD stage 4‐5 who receive sofosbuvir‐based regimens, and regular haematological blood tests (every 4 weeks) should be done in patients who receive ribavirin.

### 3.9 Monitoring of patients who achieved an SVR

The achievement of an SVR 12 is the definition of cure as persistent and lifelong HCV RNA undetectability is maintained in 99.3% of patients 37. Whether SVR patients need to be maintained on regular liver follow up is determined by the pre‐treatment fibrosis stage (advanced fibrosis/cirrhosis vs. mild/moderate fibrosis) and/or the presence of comorbidities known to have an impact on disease progression rates and HCC development (diabetes, alcohol abuse, overweight, other causes of liver diseases) 5, 9. Non‐cirrhotic patients who achieve an SVR should be retested for HCV RNA at 48 weeks post‐treatment and if HCV RNA remains undetectable, then there is no need for further follow up 5. Non‐cirrhotic patients with comorbidities known to influence the residual risk of liver cancer or known to cause liver damage should remain under regular follow up with annual blood tests and liver ultrasound.

Cirrhotic patients who achieve an SVR have been shown to have comparable survival rates to the general population matched by age and sex. However, there is a residual risk of HCC development (0.3%‐1% yearly incidence), which is influenced by age and presence of decompensated cirrhosis 38, 39, 40. Thus, patients with advanced fibrosis (METAVIR score F3) or pre‐existing cirrhosis and an SVR should remain under surveillance for HCC every 6 months by ultrasound and for oesophageal varices by endoscopy if varices were present at pre‐treatment endoscopy. The duration of HCC surveillance in these patients with advanced fibrosis or cirrhosis who achieve an SVR is indefinite 5.

In HIV‐HCV‐coinfected patients successfully treated with DAAs, considering the potential residual higher risk of progression and the lack of specific data in this population, there is still a need for close follow up following HCV cure. Another potential risk is reinfection due to persistent risk behaviour. Reported rates of reinfection following successful HCV treatment among patients at high risk are estimated at between 1% and 5% per year, higher particularly in men who have sex with men practicing “chemsex” 41. Thus the ease of Interferon free therapy may increase the likelihood of reinfection. In order to maximize the benefit of therapy, the risks of reinfection should be emphasized to patients at risk, and behavioural modifications should be positively reinforced. HCV RNA should be closely monitored in patients with continued risk practices (for example, ongoing intravenous drug using, “chemsex” and mucousal‐traumatic sexual practices).

## 4 Conclusions

The era of DAAs has revolutionized HCV therapy, with the vast majority of patients having access to treatment expected to be cured of HCV infection. Recently approved DAA combinations herald a new paradigm of shortened‐duration pan‐genotypic regimens. A number of factors pre‐therapy still determine optimal regimens, duration of therapy, the need for additional ribavirin and on‐treatment monitoring for toxicity, but this may not be required in the future as we move towards pan‐genotypic regimens. As treatments get easier in terms of adverse effects, and shorter, on‐treatment monitoring will also diminish for the vast majority of patients.

There is already a study underway to assess response without the need for on‐treatment monitoring and clinic visits for non‐cirrhotic patients (SMART‐C study ClinicalTrials.gov Identifier: NCT03117569).

Economic considerations should also not impair access to these treatments for all patients infected with HCV. Future emphasis will be identifying all these patients with HCV, increasing and facilitating access to therapy, to achieve the WHO goals to reduce incidence of new HCV infections by 90% and mortality by 65% by 2030.

## Competing interests

A Aghemo has received honoraria, fees and travel grants from Abbvie, Gilead, MSD, Janssen, BMS and research grants from Gilead. S Bhagani has received honoraria, fees, travel grants and research grants from Abbvie, Gilead and ViiV. L Piroth has nothing to disclose.

## Authors’ contributions

Alessio Aghemo, Lionel Piroth and Sanjay Bhagani designed the review, drafted the manuscript and critically revised it.
